# Detecting sorghum aphid infestation in grain sorghum using leaf spectral response

**DOI:** 10.1038/s41598-024-64841-8

**Published:** 2024-06-18

**Authors:** Grace Craigie, Trevor Hefley, Ivan Grijalva, Ignacio A. Ciampitti, Douglas G. Goodin, Brian McCornack

**Affiliations:** 1 Department of Entomology, Kansas State University, Manhattan, KS 66506 USA; 2 Department of Statistics, Kansas State University, Manhattan, KS 66506 USA; 3 Department of Agronomy, Kansas State University, Manhattan, KS 66506 USA; 4 Department of Geography and Geospatial Sciences, Kansas State University, Manhattan, KS 66506 USA

**Keywords:** Plant signalling, Agroecology

## Abstract

Sorghum aphid, *Melanaphis sorghi (Theobald)* have become a major economic pest in sorghum causing 70% yield loss without timely insecticide applications. The overarching goal is to develop a monitoring system for sorghum aphids using remote sensing technologies to detect changes in plant-aphid density interactions, thereby reducing scouting time. We studied the effect of aphid density on sorghum spectral responses near the feeding site and on distal leaves from infestation and quantified potential systemic effects to determine if aphid feeding can be detected. A leaf spectrometer at 400–1000 nm range was used to measure reflectance changes by varying levels of sorghum aphid density on lower leaves and those distant to the caged infestation. Our study results demonstrate that sorghum aphid infestation can be determined by changes in reflected light, especially between the green–red range (550–650 nm), and sorghum plants respond systemically. This study serves as an essential first step in developing more effective pest monitoring systems for sorghum aphids, as leaf reflection sensors can be used to identify aphid feeding regardless of infestation location on the plant. Future research should address whether such reflectance signatures can be detected autonomously using small unmanned aircraft systems or sUAS equipped with comparable sensor technologies.

## Introduction

The sorghum aphid, *Melanaphis sorghi* (Theobald), is a major agricultural pest of sorghum (*Sorghum* sps.) and sugarcane (*Saccharum* spp.)^1^. In the United States, sorghum aphid populations were first documented in 2013 infesting sorghum fields in Texas^2^. In the subsequent years, sorghum aphids spread into other regions of the U.S. and in 2015 were first observed in Kansas sorghum fields^3,4^. Nationwide, sorghum aphids have caused significant losses to growers as populations can grow exponentially, particularly under hot and dry conditions^4,5^. Infestations can cause up to 75–100% yield loss to growers without the timely application of an insecticidal treatment^6^.

Sorghum aphid feeding directly damages sorghum as it can cause reduction of grain quality and quantity^1,7^, delayed plant development^4^, and leaf chlorosis and death^1,8^ depending on the developmental stage and conditions in which sorghum is infested. Sorghum aphids also cause indirect damage to sorghum host plants as they excrete a clear, sticky excrement called honeydew^9^, which coats the leaf epidermis underneath active feeding sites^1^. High levels of honeydew on leaves promotes black sooty mold growth, which blocks photosynthesis^1,9,10^, and causes mechanical issues during harvest^3^.

Due to high levels of plant damage and the potential for rapid population growth, frequent field scouting is needed to control sorghum aphid outbreaks in sorghum fields^11^. Growers and scouts must locate sorghum aphid infestations manually, estimate populations, and determine when plants need to be treated with insecticide based on economic threshold levels. However, physically walking through fields to visually locate populations is not time efficient and often impractical for detecting crop pests^12^, especially for farmers with high-acreage sorghum fields (e.g., > 1000 acres). Our research goal is to help overcome these monitoring limitations by developing a more efficient monitoring system by using remote sensing technologies. Remote sensing equipment measures wavelengths of light reflected off leaves that relays information about the overall “health” or plant reponses to light^13,14^. Plants with environmental stress will undergo different physiological changes, based on the type of stressor, which alters light reflection patterns compared to unstressed plants^12,15,16^. This leaf spectral response is due to internal physiological changes by the plant that which include altered leaf pigment content and tissue structure^17,18^. These physiological responses are correlated with reflection changes in the visible or near-infrared (NIR) regions of the electromagnetic spectrum, respectively, that can provide a diagnosis into the physical and chemical state of the stressed plant (i.e., “health”) based on detectable changes in key or diagnostic wavelengths^15,16^. This physiological-spectral response is the foundation for diagnosing crop stress, as these measurements can be taken multiple times without damaging plant tissue and using sophisticated sensory technologies^12^.

Airborne remote sensing technology, such as uncrewed aircraft systems (UAS), has been used to capture leaf spectral data from agricultural fields and such systems can provide timely and cost-effective data about field-wide plant health^19,20^. However, the exact leaf reflectance response can vary between crop systems based on the type of environmental or biological stressor, variations in internal leaf structures, and growing conditions during development^15,21–23^. As leaf spectral responses can vary widely, several key variables such as spectral signature, aphid density needed to elicit a physiological response, and local versus a systemic response, need to be addressed before implementing a sorghum aphid monitoring system to assure populations are being detected accurately and efficiently.

One of the first steps to developing a UAS-based monitoring is to determine the signature spectral response or specific changes in wavelength reflection due only to sorghum aphid stress in sorghum. These precise changes in wavelength ranges are not currently known, making it difficult to distinguish sorghum aphid feeding from similar insect pests and determine what physiological effects sorghum aphids have on sorghum, such as altered leaf pigment content or cell structures. In addition, the density level of sorghum aphids needed to elicit a detectable leaf spectral response also needs to be studied as aphid populations can grow exponentially and therefore needs to be detectable at low densities. In this research, we assessed reflectance changes to sorghum aphid feeding on sorghum under greenhouse conditions. It is currently unknown if sorghum initiates a localized (i.e. near the feeding site), or systemic response (i.e. plant-wide) to sorghum aphid infestation in a way that can be detected using leaf spectral response. This knowledge gap poses a problem in implementing UAS for sorghum aphids as they tend to feed on the underside of lower canopy leaves^24^, whereas UAS take spectral readings from upper canopy leaves^25^. If sorghum aphid feeding response is only localized, then populations feeding on lower leaf levels may not be detectable using a UAS. A measurable systemic response to sorghum aphids is needed to define the location of aphid infestation does not disrupt the efficiency of remote sensors.

Based on previous studies that have successfully detected aphid feeding using remote sensing in agricultural crops^12,26–28^, we hypothesize that sorghum aphids, at low-density levels, will cause enough physiological changes to sorghum to be detectable using remote sensing technology. In addition, our results are likely to show both a local and systemic response to sorghum aphid feeding as a similar aphid species, greenbugs (*Schizaphis graminum*), has been demonstrated to induce defense pathways in sorghum^29^. If the presence and feeding of sorghum aphid on a lower leaf, for example, induces a systemic defense pathway than enough physiological alteration throughout the plant should occur to allow aphid infestation to be detectable regardless of infestation sites.

The overall objective of this study was to analyze changes in sorghum response to sorghum aphid feeding by comparing leaf reflectance between infested and non-infested plants. This will hypothetically allow us to discern sorghum plant’s signature leaf spectral response to sorghum aphid feeding by measuring spectral reflectance near active feeding sites. Additionally, to determine if changes in sorghum response to sorghum aphid feeding is to be only detectable locally or systemically using remote sensing technology.

## Methods

### Sorghum aphid infestations

Sorghum aphids used for the study were wild caught from naturally infested sorghum fields during the summer of 2016 and 2017. Apterous aphids were collected from fields within the Ashland Bottoms and North Research Farm in Manhattan, KS. Fine-tipped paintbrushes were used to transfer aphids from infested plants to Eppendorf tubes. Aphids were then infested to potted sorghum plants (DKS29-28, Dekalb) under greenhouse conditions. Newly infested plants were placed into Bugdorms (cage-2400, BugDorm; Taiwan) rearing tents and all sorghum aphid colony Bugdorms were placed in the same greenhouse space. Prior to infestation, DKS29-28 sorghum plants were grown in a separate greenhouse room from aphid colonies to prevent unwanted infestations. Sorghum variety, DKS29-28 (Pioneer, Corteva Agriscience, Iowa, U.S.A.), is an early-maturity hybrid that is considered susceptible to sorghum aphids.

All potted plants were grown using Sungro Professional Growing Mix (SunGro; Agawam, MA). Seeds were first planted in cone-shaped pots (dimensions: 3.81 cm diameter × 20.955 cm depth × 238.91 ml volume), referred to as “Cone-tainers” (model SC10, Ray Leach Cone-tainers, Tangent, OR) with 18 cm of soil per pot. All sorghum was grown in a greenhouse under artificial lights with 16:8 h. (light: dark) photoperiod, maintained at 22 ± 3 °C, fertilized with “Peter’s Professional 20-20-20 General Purpose Fertilizer,” and were regularly inspected to ensure that no aphid or other insect pests were present. Sorghum plants were allowed to grow until they reached 3-leaf stage or Vegetative Stage 1. Plants were then transplanted into 15.24 cm diameter plastic pots, to provide adequate room for growth, and placed in clean Bugdorms with no prior contact with aphids. All plants were grown to the 5–7 leaf stage or Stage 2 before being used for greenhouse experiments. Sorghum plants in the 5–7 leaf stage were selected to accommodate the width of the clip cage, meaning they needed plenty of height for growth.

Sorghum aphid colonies were kept in Bugdorms to contain colonies, and prevent unwanted infestation to other greenhouse spaces, and were regularly supplied with sorghum plants. For rearing aphid colonies for use in further experiments, high numbers of sorghum plants were needed to maintain colonies between 2016, when aphids were first collected, up through 2018 when the experiments took place. Both plant-rearing and aphid colony rooms were maintained under similar greenhouse conditions under artificial lights at 16:8 h. (light: dark) photoperiod and maintained at 22 ± 3 °C temperatures throughout the year. Sorghum aphid voucher specimens, *Melanaphis sorghi* (Theobald) apterous and alate adults, were deposited in the KSU Museum of Entomological and Prairie Arthropod Research Department (voucher # = 263).

### Greenhouse research experiments

**Figure 1:**
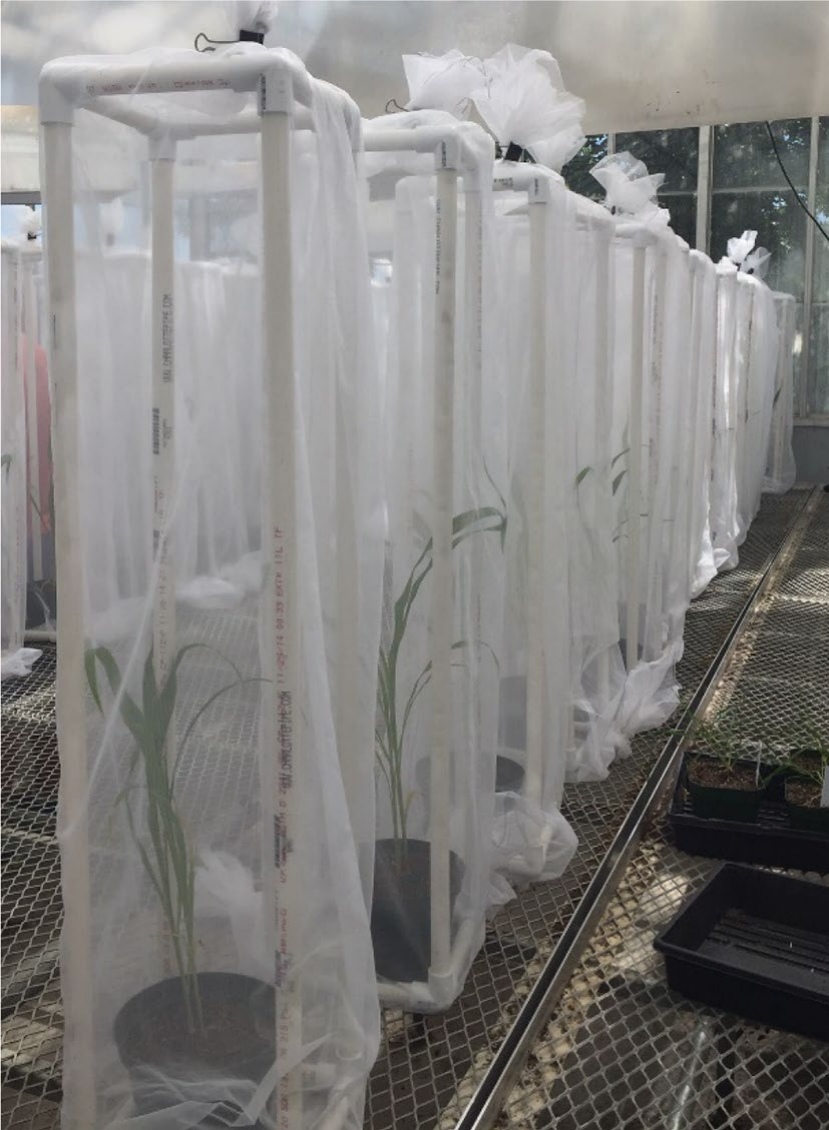
Exclusion tents with dimensions: 21.59 × 21.59 × 96.52 cm.

**Figure 2:**
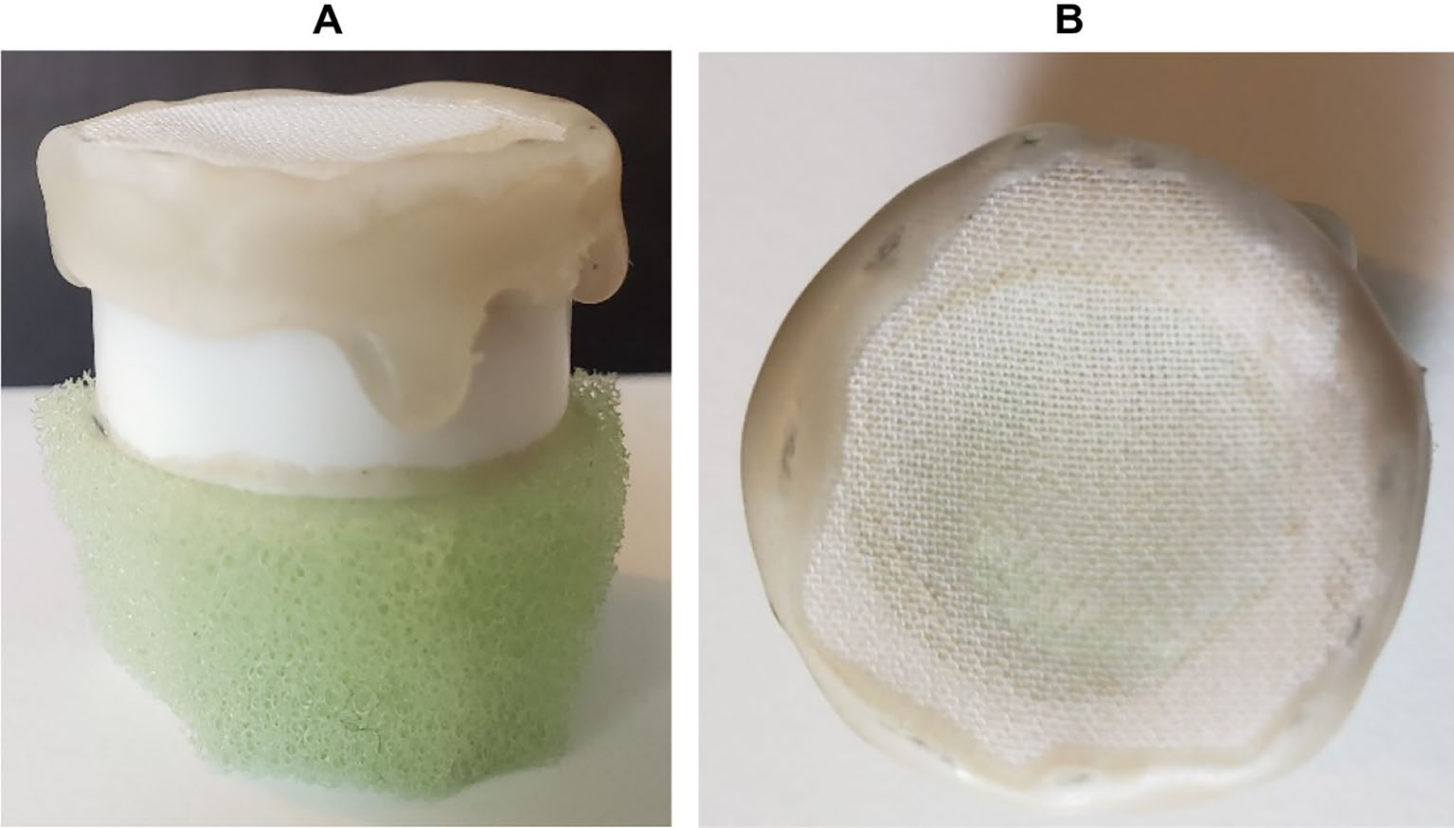
Clip cage used to house sorghum aphids. (**A**) Side view and (**B**) to view of clip cage. with dimensions: 2 × 2 × 2.5 cm.

**Figure 3:**
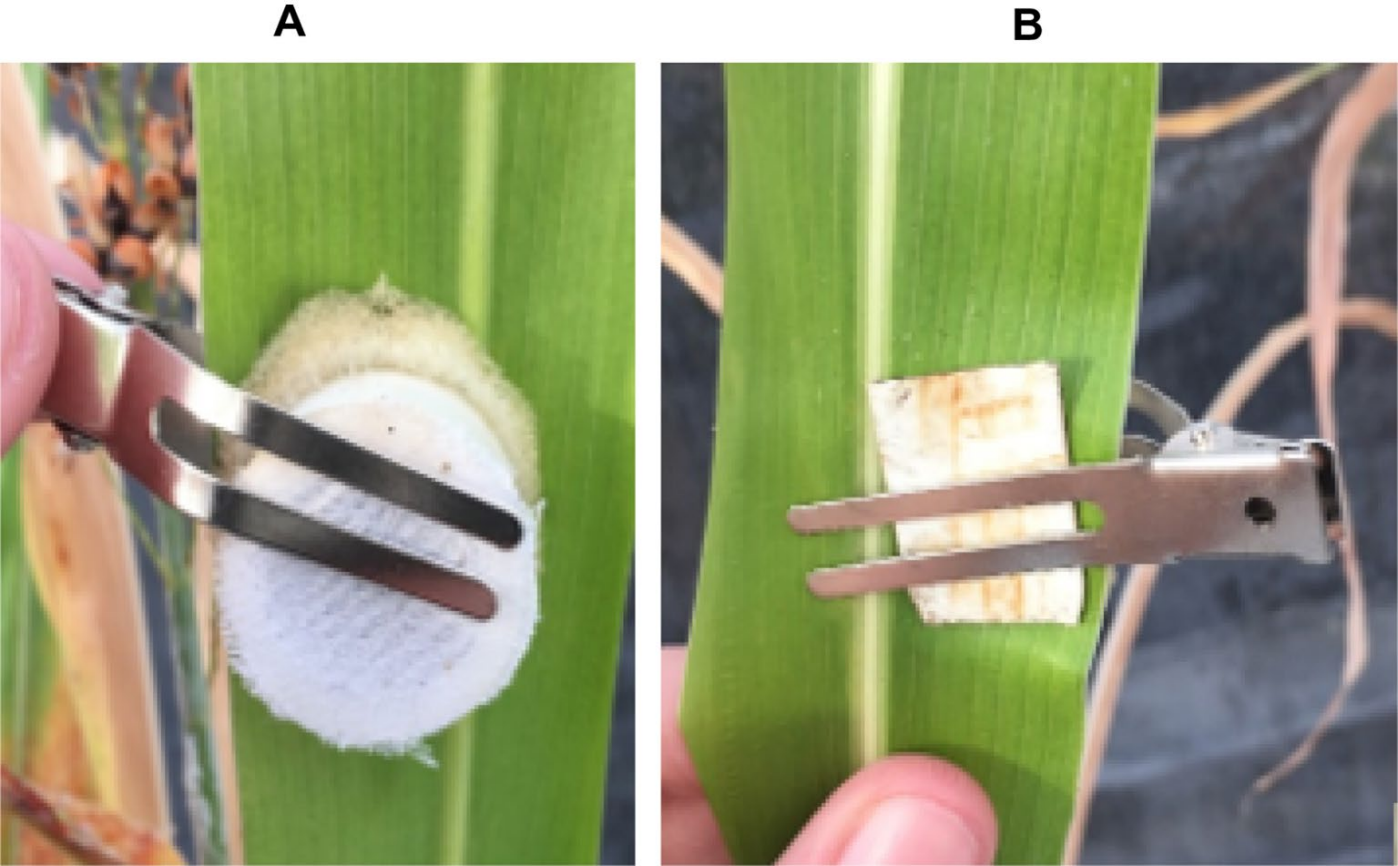
Clip cage attached to a sorghum leaf from view (**A**) and (**B**) back view.

Each run of the greenhouse study was set up in a randomized complete block design and was carried out 14 days post initial aphid infestation. Sorghum pots were placed in 32 mesh SL2.0 exclusion tents (dimensions: 21.59 W × 21.59 L × 96.52 H cm) created from cut pvc pipe pieces and pvc pipe joints, wrapped in sewed mesh sleeves, and had the top sealed with a binder clip (Fig. 1). Each tent had 1 sorghum plant pot per tent and were randomly assigned different treatments of 0, 2, or 10 apterous, adult sorghum aphids. As sorghum aphid feeding needed to be measured near active feeding sites, both for determining a signature spectral response and to analyze local versus systemic sorghum responses, aphid movement was restricted by housed aphids in clip cages. Clip cages (dimensions: 2W x 2L × 2.5H cm) were created with a cut piece of pvc pipe surrounded on the bottom by a ring of foam for padding and with the top covered in mesh for ventilation (Fig. 2). Each cage was secured to the leaf using a metal utility clip with a plastic backing placed between the metal clip and the leaf to decrease chance of tissue damage (Fig. 3).

In addition, rods with metal loops were attached to each clip cage to support the weight of the cage. All clip cages were positioned on the abaxial side of the leaf as sorghum aphids tend to feed on the underside of leaves. In addition, all clip cages for the experiments were placed in the middle section of the leaf and at the third leaf position from the bottom, as this leaf is typically sampled during scouting, and position was kept consistent for all replications. The third leaf position from the bottom because lower, older leaves senesced during pre-tests within the duration of the experiment. Using clip cages to house aphids also played into the size of the sorghum plants used in each run of experiment. Clip cages have a 2 cm width which means that sorghum leaves needed to have a width > 2 cm to prevent aphid escape and ensure that all aphids were given equal access to leaf tissue.

In preliminary experiments, sorghum plants that were in the 5–7 leaf stage consistently had leaves wide enough to encompass the clip cages and were less likely to exhibit structural damage when the clip cage was secured to the leaf. Smaller leaves had higher chances of damage due to the mechanical pressures of the clip cage and metal clip used to secure the cage. Therefore, all plants used in each experiment were in the 5–7 leaf stage as leaves were wide enough for the clip cage. Although using clip cages are important to restricting aphid movement, cage effects on leaves was expected to elicit a physiological response from sorghum for several reasons; added shading, temperature and humidity differences, and the chance that the metal clip used to secure the clip cage could cause minor, mechanical damage to leaf tissues.

**Figure 4:**
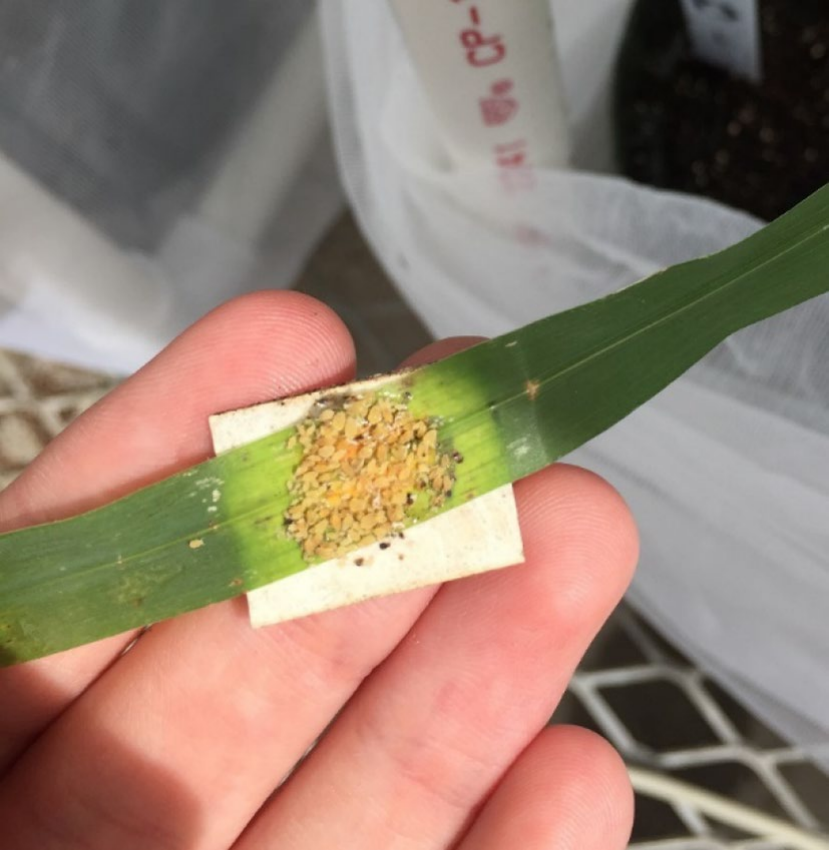
Example of population growth of sorghum aphid within an exclusion clip cage.

In our study, two control groups (i.e. non-infested treatments) were used to account for cage effect on sorghum leaves. The first control (C1) had no clip cage, and no aphids present on the plant to provide a baseline comparison between infested and non-infested plants. The second control (C2) had an empty clip cage on the plant with no sorghum aphids. C2 was added to measure any stress, and potential leaf spectral response due to the clip cage, which could be distinguished from stress due to sorghum aphid feeding. In addition to the two control groups, two aphid infestation treatment groups were tested. To determine the density of sorghum aphids needed to elicit a leaf spectral response, two different starting densities of aphids, low and high, were used by infesting sorghum leaves. For the low aphid density treatment (T1), only 2 adult, apterous aphids were initially placed in each low-density clip cages. Conversely, high aphid-density cages (T2) started with 10 adult, apterous aphids. Both aphid groups in T1 and T2 aphid populations were allowed to reproduce within respective clip cages throughout the duration of each experiment (Fig. 4).

### Measure of spectral response

**Figure 5:**
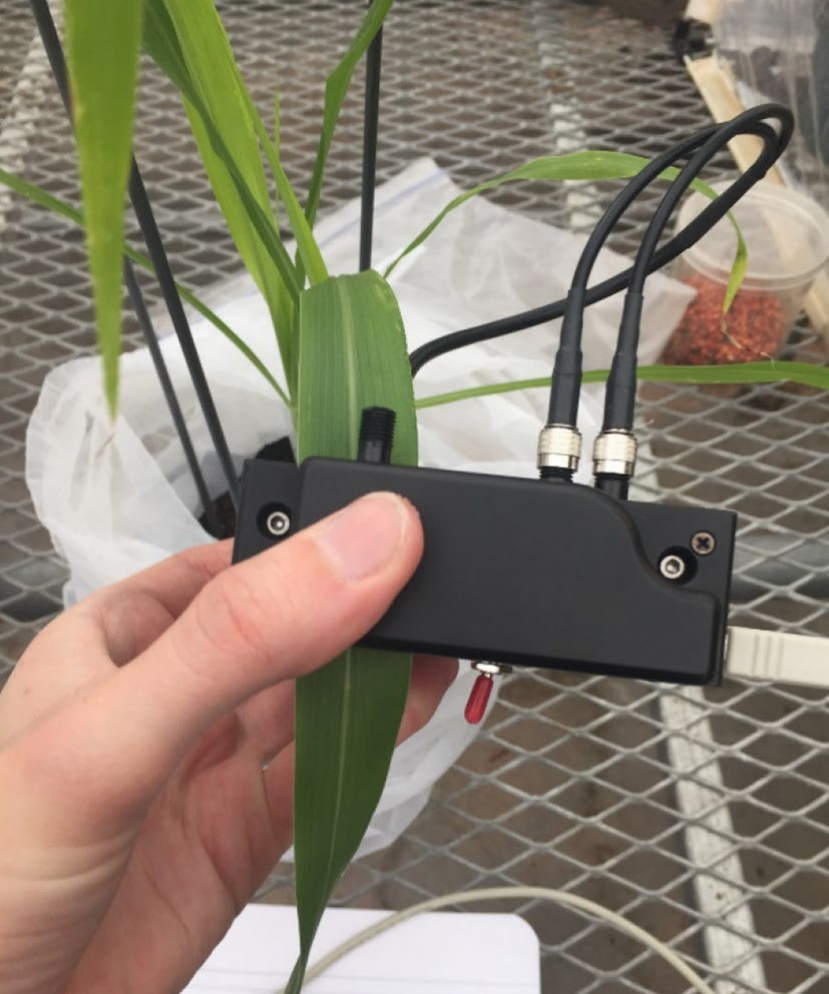
Measuring leaf reflection on a sorghum leaf using a CI-710 miniature leaf spectrometer.

Once the experiment was set up in the greenhouse, light reflectance was measured 24 h. after initial aphid infestation (Day 1) and continued once a day for 14 days post initial infestation (Day 14). Leaf spectral response on individual sorghum leaves was measured using a hand-held CI-710 Miniature Leaf Spectrometer (SpectraVue Leaf Spectrometer, CID Bio-Science) (Fig. 5). All spectral readings were taken in the morning starting around 9:00 am for a 2 h period, and always starting with control tents (C1 and C2) and then the aphid treatment groups (T1 and T2) to prevent accidental infestation of sorghum aphids to control tents. The spectrometer uses a broadband light source and measures reflectance in the visible and near-infrared (NIR) wavelength ranges, between 400 and 1000 nm.

**Figure 6:**
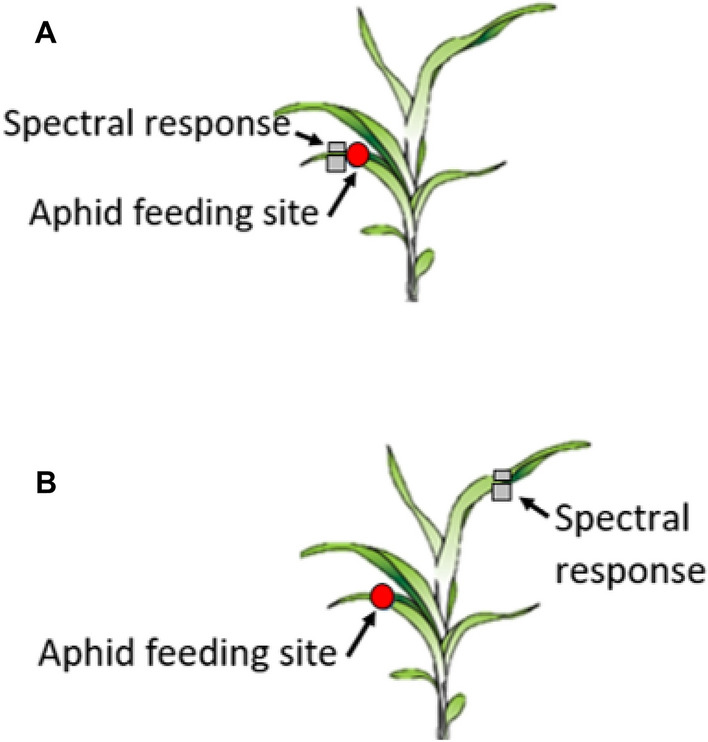
Leaf spectral reflectance measurements are taken in relation to location of active aphid feed (in clip cage). (**A**) Spectral measurements near active feeding site. (**B**) Spectral measurements on distal leaf from active feeding site.

This wavelength range is analogous to hyperspectral sensors currently available for remote deployment using small UAS. For the first run of the experiment (R1), the objective was to detect sorghum aphids near the feeding site, next to the clip cage (Fig. 6A). The leaf spectral response of sorghum over time, as aphid population increased, was also analyzed. The second (R2) and third runs (R3) also measured spectral response near the active feeding site and how spectral response may have been affected over time.

However, an additional variable was added to these two runs as spectral measurements were also taken at a distal location, on a different non-infested leaf, from aphid infested cages to see if the presence of sorghum aphids could be detected systemically (Fig. 6B). Local spectral measurements, labeled A position, were taken near clip cages for the C2, T1, and T2 treatments. Measurements were consistently taken directly next to the clip cages on the side of the leaf near the petiole. A second reading, labeled D position, was taken distal to the feeding site. This was done by taking a reading that was two leaf positions from the clip cage towards the newest leaf. Since cages were placed on the third leaf position, distal readings were taken on the fifth leaf. All treatments, with or without clip cages/aphids, measurements were taken in the same location of the middle and abaxial side of the sorghum leaf for consistency’s sake. In addition, control tents (C1 and C2) were always measured before treatment (T1 and T2) tents to reduce the chance of unwanted sorghum aphids dispersing to control tents.

### Statistical analysis of experimental data

We jointly analyzed all three replicates (i.e., R1, R2, and R3) of the experiment to determine the influence of sorghum aphid infestation on sorghum leaf reflectance using a machine learning approach known as boosted regression trees^30,31^. This machine learning approach enables prediction of the expected leaf reflectance in the visible wavelength range of 500–799 nm, green to red light, for sorghum plants at both the feeding site and distal location between 1 and 14-days post infestation under the two treatments (T1 and T2; low and high aphid density) in addition to the two controls (C1 and C2; no aphids without cage and no aphids with cage).

A range of only 500–799 nm was used for this study due to limitations in the CI-710 Miniature Leaf Spectrometer at low and high reflectance readings. The output for this analysis includes heatmaps that show predicted expected leaf reflectance. Like any map (e.g., geographic map), there is an x-coordinate and a y-coordinate that determine the “location” of a point. For our analysis, the x-coordinate is the day since aphid infestation and the y-coordinate is wavelength. That is, for a chosen “location” (i.e., a specific day and wavelength) a heatmaps provides a prediction of the expected leaf reflectance. Furthermore, since there are two aphid densities (T1 and T2), two controls (C1 and C2) and two points of measurements (adjacent and distal to feeding site), a total of eight heatmaps were generated.

To infer the treatment effects, we compared the predicted expected reflectance among the eight heatmaps. The difference between the predicted expected reflectance for a chosen comparison was used as an estimate of the main effect. For example, we compared the predicted expected reflectance for T2 to the predicted expected reflectance of T1 to estimate the effect that aphid density had on leaf reflectance. As another example, we compared the predicted expected reflectance for C2 to C1 to determine the impact of cages on leaf reflectance. Similar to our heatmaps, a one-dimensional visualization of all predictions are reported. The one-dimensional visualization shows the predicted leaf reflectance for any given sample date for a pre-selected wavelength.

Our “predictive approach” to infer the effects aphid infestation on sorghum leaf reflectance departs from traditional approaches used for designed experiments (e.g., hypothesis testing using analysis of variance). There are two important reasons why a predictive approach was selected in addition to at least one important benefit. The first reason why a predictive approach was used is that treatment affects are likely to have complex dependence on the interaction between wavelength and day since day since aphid infestation. While traditional approaches such as analysis of variance do enable the quantification and testing of interaction effects, the major drawback is the need to conduct for every wavelength and day combination for each of the eight combinations of treatment, control and location of measurement; this is clearly infeasible because the number of comparisons is too large for a human to interpret. Boosted regression trees are ideally suited for detecting and quantifying high-order interactions like those mentioned above^30^.

The second reason a predictive approach was used is that the data set is rather large (i.e., > 450 K responses) and with near certainty all treatment, control, and location effects would be “statistically significant” using traditional hypothesis testing, using p-values for example^32^. As a results, the magnitude of the effect (e.g., what is the impact on leaf reflectance of having a high vs. low aphid density on day 12 at a wavelength of 603) would still need to be quantified. Thus, this innovative approach circumvents the need to find “statistical significance”, which almost surely can be found in a large data set, by using modern, predictive approaches that are well-equipped and designed to quantify such effects. Finally, there is at least one benefit to a predictive approach. In the next paragraph, we explain how we validate our predictions. Because of this validation step, we were able to produce heatmaps (and other predictions) that quantified the reflectance for a given day and wavelength that have an assessed level of accuracy. Like other types of maps (e.g., geographic), it is important to quantify and communicate their accuracy. Finally, and regardless if the method of analysis is traditional (e.g., analysis of variance) or modern, it is important to validate the analysis, which typically requires either a second data set or a large data set that can be split into two with one portion reserved for validation.

As mentioned above, boosted regression trees were used to analyze data from all three replicates (i.e., R1, R2, and R3) of our experiment. Boosted regression trees are a machine learning approach that are known to have high predictive accuracy and are capable of detecting and quantifying high-order interactions. We chose to use boosted regression trees because we desired that our predicted heatmaps be as accurate as possible and because we anticipated complex interactions among the predictor variables day, wavelength, treatment, control and location. We split our data into a training and validation set by randomly allocating 50% of the observations to each set. This resulted in 276,151 observations in the training data set and 276,151 observations in the test data set. We then fit multiple boosted regression tree to the training data set using different combinations of the predictor variables (Table 1).

**Table 1:** Predictive variables and scores day post infestation (Day), wavelength (wl), treatment group (TRT), proximity to the aphid feeding site (Prox), and plant ID (pid).

Predictor variables	Predictive error
None	0.136238
Day + wl	0.022152
Day + wl + TRT	0.020739
Day + wl + Prox	0.021395
Day + wl + TRT + Prox	0.020168
Day + wl + TRT + Prox + pid	0.014966

We fit our boosted regression trees using the gbm package in program R using a Laplace distribution (i.e., absolute loss function), a total of 100 trees, an interaction depth of 30, a bag fraction of 1.0 and a shrinkage rate of 0.1. To quantify the predictive accuracy of each boosted regression tree, we predicted the expected leaf reflectance for all observations, all control and treatment groups, in the validation data set. We then calculated the absolute difference between the recorded leaf reflectance and the predicted reflectance. We then took the average of this difference (i.e., the average predictive error) across all 276,151 observations in the validation data set. When evaluating predictive performance, it is important to have “dummy” metrics for comparison (i.e., a method of prediction that is simple to obtain that has some predictive accuracy). As such, we used the average leaf reflectance obtained from the training data set, which was 0.1769. Using this value of 0.1769 as a predictor for all 276,151 leaf reflectance measurements in the validation data set results in a predictive error of 0.1362. That is, the average absolute difference between the recorded value of leaf reflectance and the predicted (using 0.1769 for all predictions) was 0.1362.

### Research statement

The plant species used for the study have permission and comply with the IUCN policy statement on research involving species at risk of extinction and the convention on the trade in endangered species of wild fauna and flora. This study complies with relevant institutional, national, and international guidelines and legislation.

## Results

### Gradient boosted regression trees

**Figure 7:**
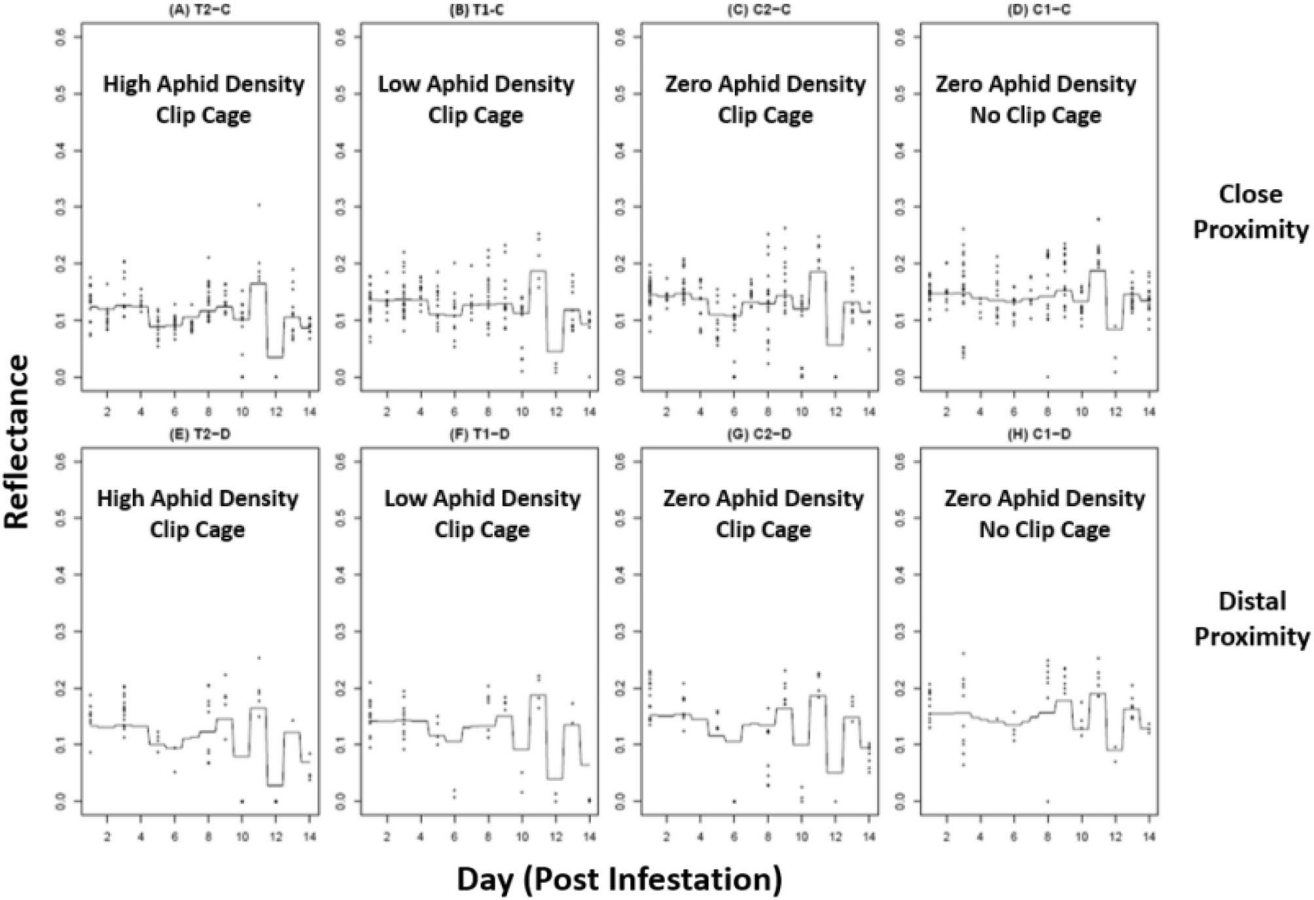
Gradient boosted regression tree at 550 nm.

**Figure 8:**
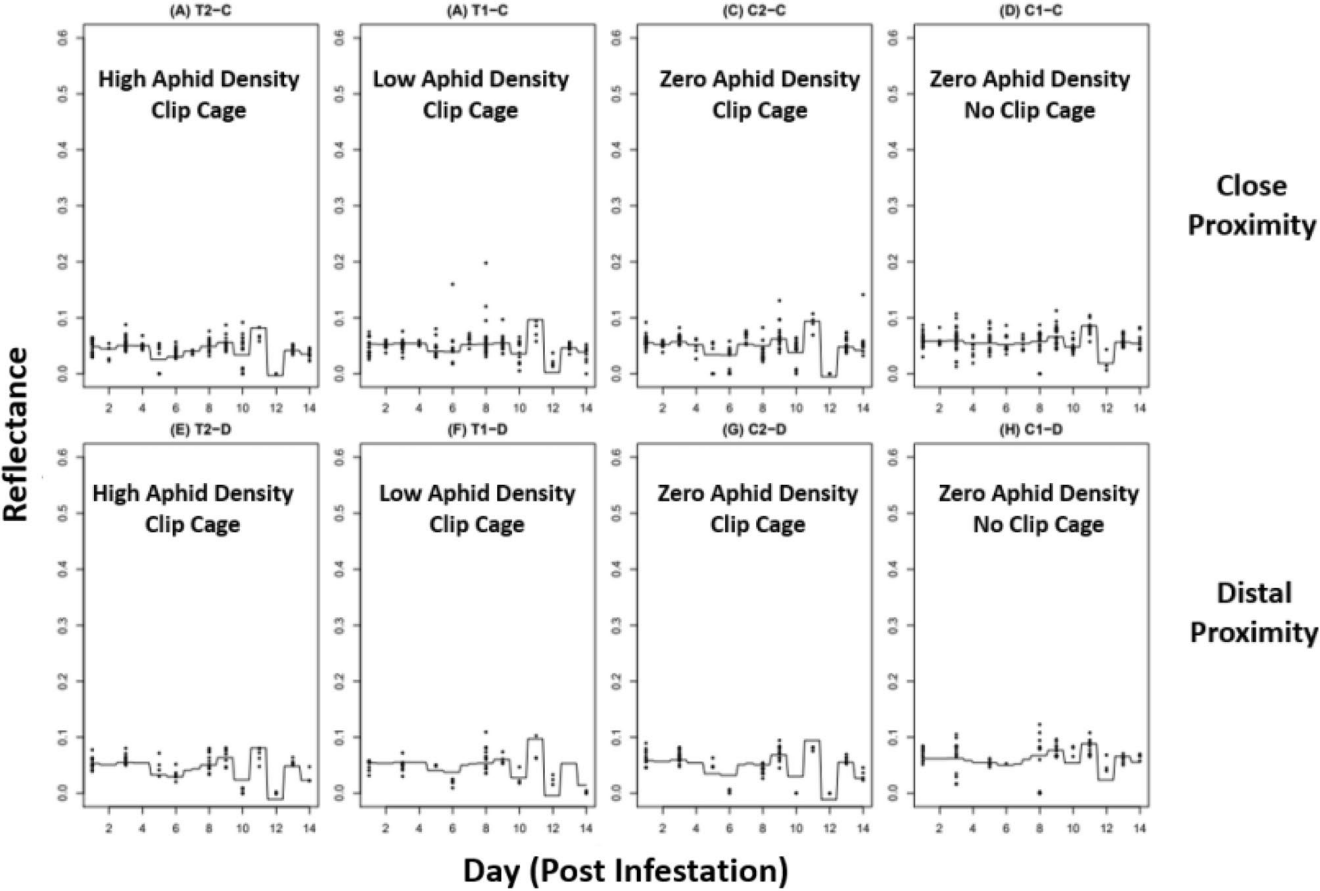
Gradient boosted regression tree at 650 nm.

**Figure 9:**
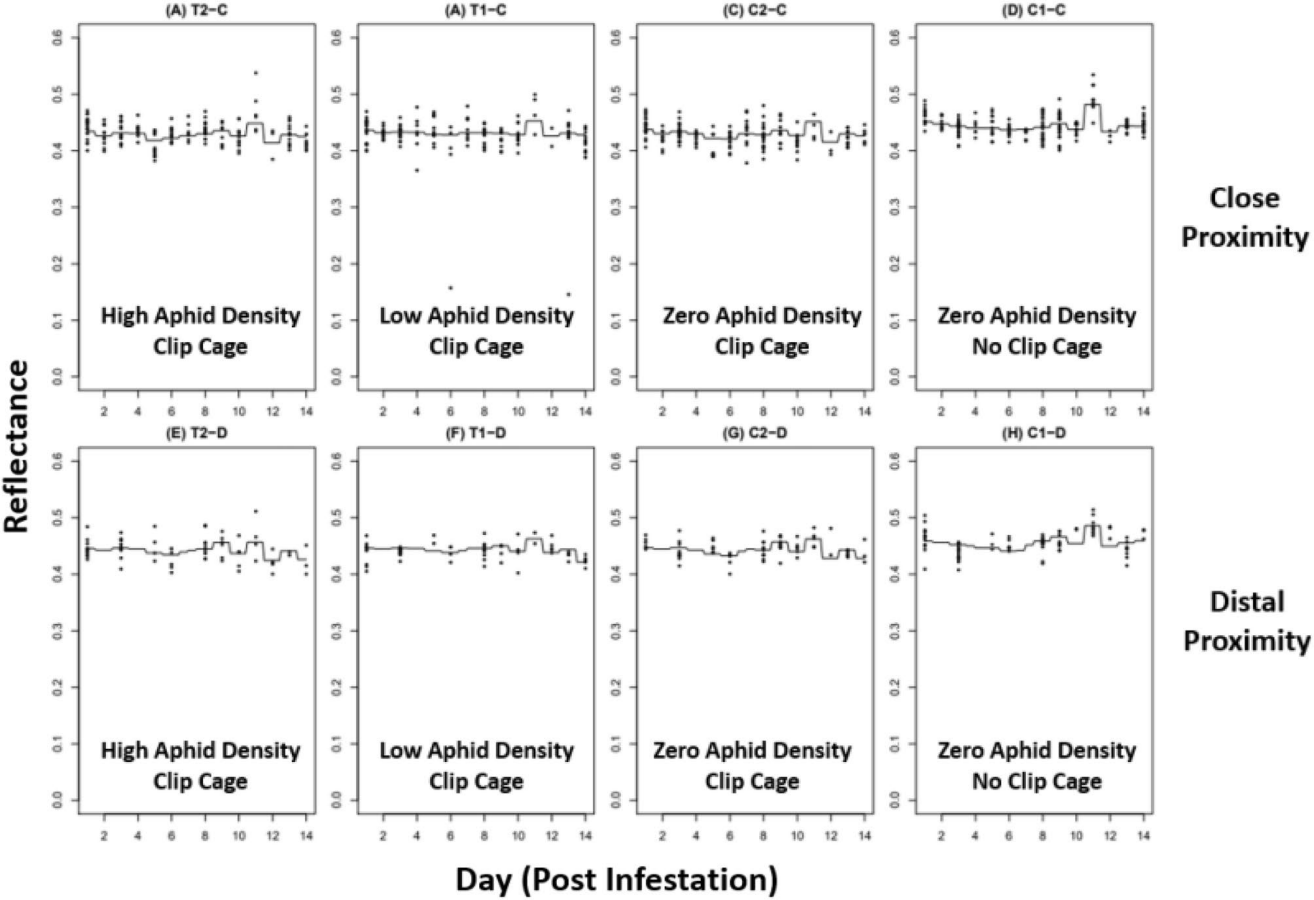
Gradient boosted regression tree at 750 nm.

Since the analysis generated a regression tree for every tested wavelength, three example wavelengths of 550, 650, and 750 nm (Figs. 7–9 respectively) were chosen to represent a cross-section of the data. The gradient boosted regression tree curves depict the predicted changes in leaf reflectance across a 500–799 nm wavelength range (Figs. 7–9). Eight maps, or panels, depict 8 different experimental groups (A-H) that were generated for each wavelength within the tested wavelength range. The top panels of each map (A-D) show the close aphid treatments of T2-C and T1-C (high and low aphid densities, respectively) and control groups C1-C and C2-C (no cage-no aphid and cage-no aphid, respectively). The bottom panels (E-H) show the distal aphid treatments of T2-D and T1-D (high and low aphid densities respectively) and control groups C1-D and C2-D (no cage-no aphid and cage-no aphid, respectively).

The changes in leaf reflectance between each day and wavelength are visibly complex as seen in the uneven and convoluted curves within each panel. This shows the intricate interactions between day and wavelength which indicates that the predicted reflection is very accurate. When comparing the 550, 650, and 750 nm regression trees, several notable differences can be detected. The curve from Days 2–10 shows a dip around Day 4 within every experimental group except the C1 (no cage-no aphid) controls. The C1 curve, in both close and far treatments, show a relatively consistent reflectance across all 3 representative wavelengths. In addition, the level of reflectance in the 550 and 650 nm (Figs. 7, 8 respectively) are comparatively lower than the 750 nm (Fig. 9), which is consistent with the spectral reflectance curve for plants. All three wavelengths also had an unexpected curve pattern that was seen across all the panels. At Day 10 there is a relatively large increase in reflectance followed by an equally large decrease in reflectance at Day 12. Interestingly, this Day 10–12 pattern is the most pronounced at 550 nm, followed by 650 nm, and then 750 nm where the pattern is less noticeable.

The gradient boosted regression trees show not only the complexity of the predicted reflectance in relation to day and wavelength but also similar sorghum response patterns between proximity treatments (i.e., close or distal to feeding site). All wavelengths were plotted, but only select ranges were included to show differences in response through time. In each example wavelength of 550, 650, and 750 nm, the close (-C) and distal (-D) locations from aphid infestation sites displayed analogous reflection curves. For example, in the 550 nm wavelength example (Fig. 7) the curve pattern during Days 2–10 were seen in both close (A-D) and distal panels (E-H). Both proximity treatments showed an unique pattern of having a flatter curve for C1 panels compared to C2, T1, and T2. In addition, the dramatic dip in reflectance around Day 10–12 was also witnessed in both close and distal within each example wavelength. Considering the complexity of the reflection curves, it is extraordinary that these patterns are consistently apparent between both close and distal groups within each regression tree example.

### Predicted reflectance heatmaps

**Figure 10:**
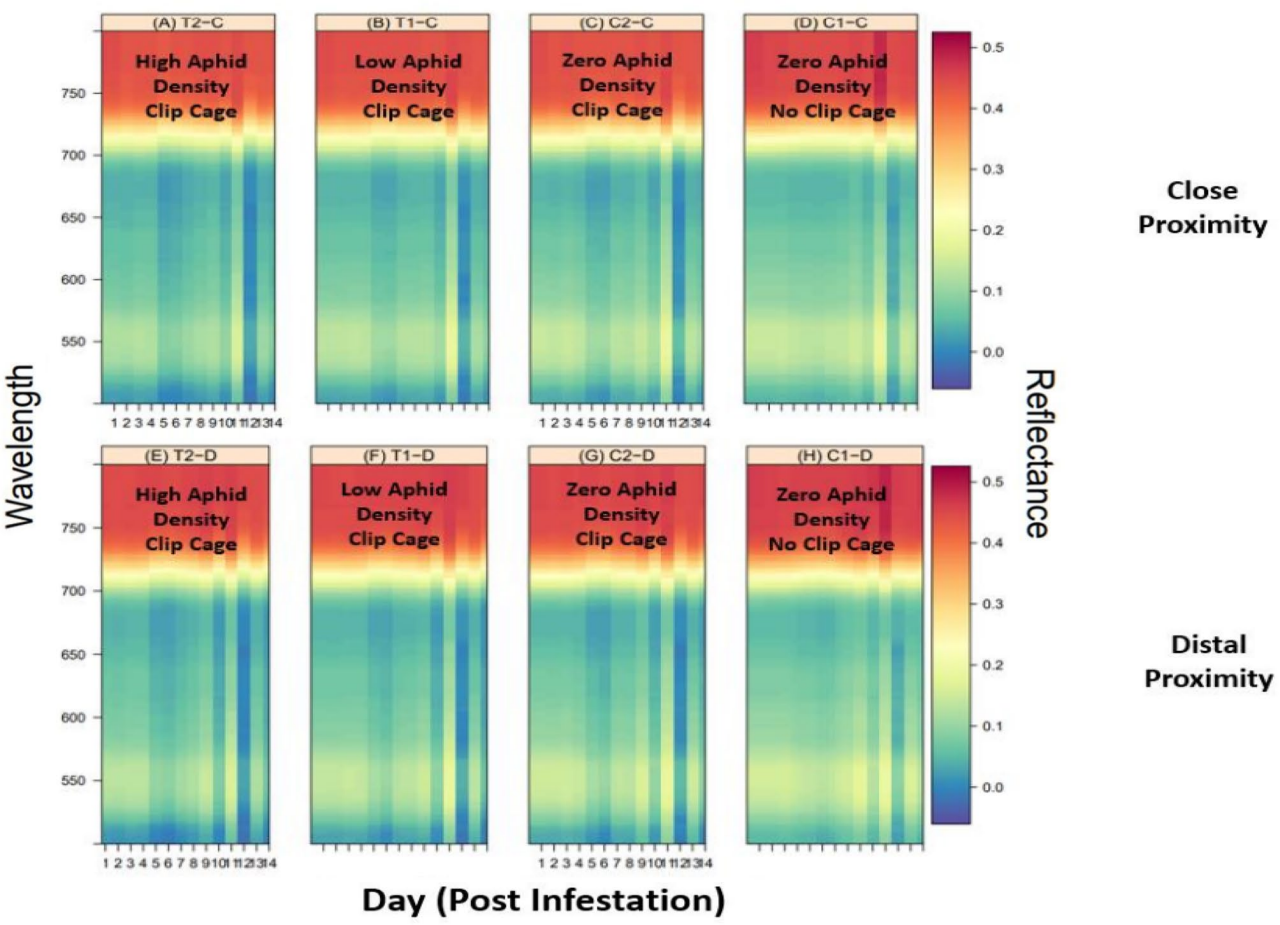
Predicted reflectance heatmap.

Another way of depicting the gradient boosted regression trees is to create a heatmap that shows day, wavelength, and reflectance (Fig. 10). This map shows predicted reflectance given a specific day and wavelength. That means that knowing how long sorghum has been infested with sorghum aphids allows one to estimate the leaf reflectance at a given wavelength. Fairly minor effects can be detected since this map was created using a very large data set. The heatmaps (Fig. 10) also show a consistency between close (-C) and distal (-D) patterns, with C1 showing a lower predicted reflection around Day 2–10 as compared to C2, T1, and T2 panels, but the intricacy of the heatmaps makes it difficult to visually discern additional similarities between proximity treatments.

### Predicted scores

Both the gradient boosted regression tree and heatmap show the complex relationship between day and wavelength in predicting reflectance. However, these are predictive maps, which introduce the question of how accurate they are in predicting leaf reflectance. As described in the methods section, predictive scores were calculated to determine accuracy (Table 1). Essentially, this table shows how accurate we can get in estimating leaf reflectance based on how many variables are known. If we take the average reflectance from all three experiments (R1, R2, and R3), you get a mean reflectance of 0.1769. From that value, we calculated a predictive error of 0.1362. In other words, if no predictive variables, such as day, wavelengths, treatment, or proximity of the leaf spectrometer to aphid feeding, are known then our prediction of reflection will be off by 0.1362.

Using the validation data set to assess predictive accuracy, we found that a boosted regression tree that uses only day and wavelength as predictor variables can predict leaf reflectance with an average error of 0.0222. By comparison if no predictor variables are used to obtain predictions (i.e., we use the value 0.1769 to predict leaf reflection regardless of the day or wavelength), we get an average error of 0.1362. This indicates that knowing the day and wavelength enables a massive improvement in our ability to predict leaf reflection; of course, this result was expected, by comparison, when we add the treatment (T1 and T2) and control (C1 and C2) as predictor variables in addition to day and wavelength, we obtain an average error of 0.0207. While the predictor variables treatment and control did not result in a major improvement in the average predictive error, we do note that this is roughly a 7% improvement in predictive accuracy compared to when the treatment and control predictor variables are not included (i.e., (0.0222 − 0.0207)/0.0207 = 0.0725).

The addition of location (i.e., feeding site or distal) as a predictor variable resulted in similar but slightly smaller increases in predictive accuracy (Table 1). Finally, the most accurate approach was the boosted regression tree that contained all predictor variables (i.e., day, wavelength, treatment, control, and location), which had a predictive error of 0.0202. These predictive scores show both the value of knowing additional variables in increasing estimation accuracy and that the system is highly predictable. As our reflection range is between 0.0 and 0.5, having an average error of only 0.0202 is very low by comparison.

### Predicted difference in reflectance

**Figure 11:**
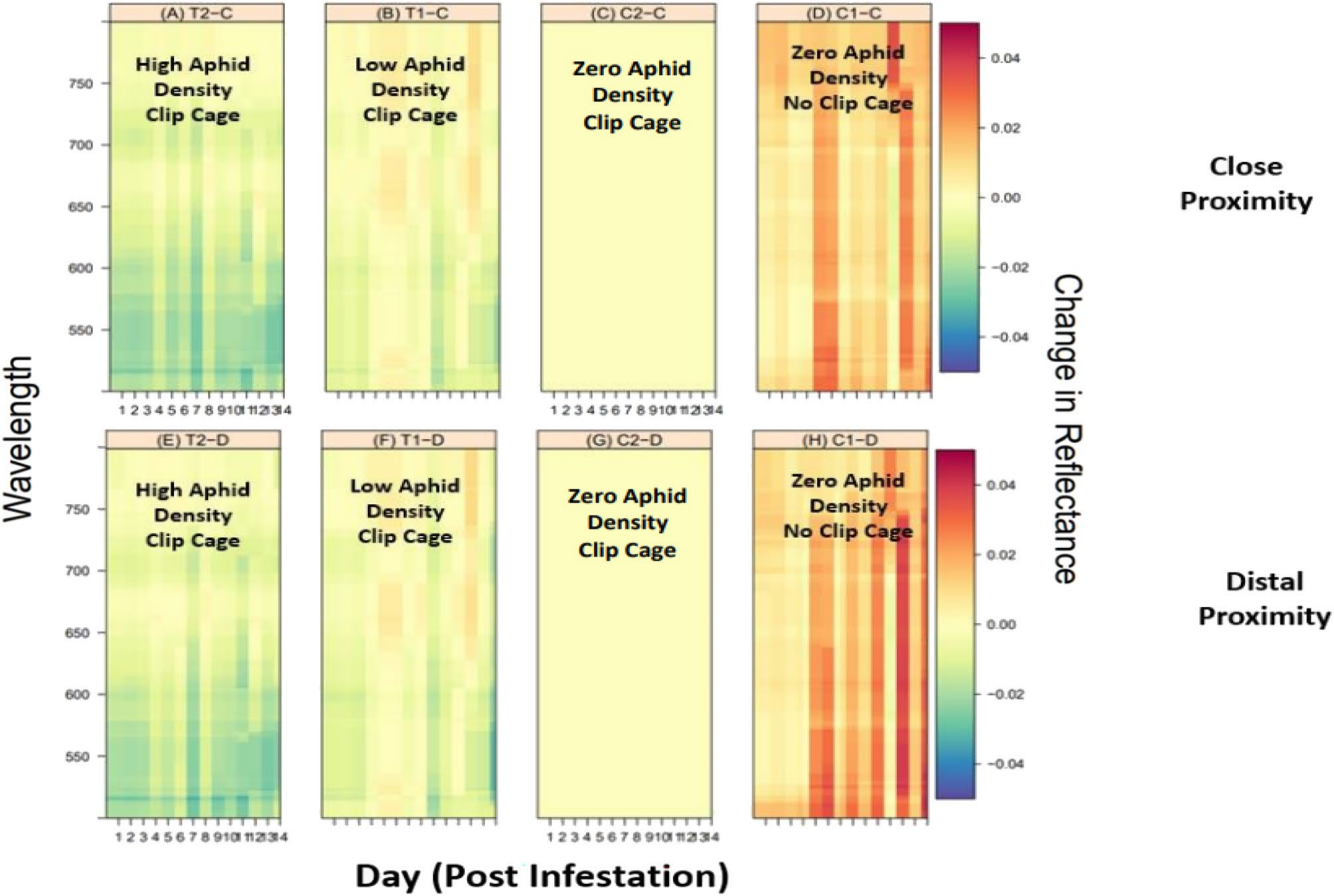
Predicted difference in reflectance between treatments.

Since the regression trees were shown to have high predictability, with only a predictive error of 0.0202 when all variables are known, we can now show that they are very accurate in determining leaf reflection. However, this high accuracy includes having a clip cage in 3 out of 4 experiment groups (C2, T1, and T2). One of the questions surrounding using clip cages to house aphids is the effect the cage might have on leaf reflectance. To tackle the potential imposition of a cage effect, we removed the experimental group C2 (cage-no aphid) from all other groups (C1, T1, and T2) (Fig. 11). The results showed a stark difference between the C1 control (no cage-no aphid) and the two aphid densities treatment groups (T1 and T2). Surprisingly, we found that in the presence of sorghum aphids (T1 and T2), the change in reflection was visibly lower than in non-infested plants (C1). In other words, in the absence of a clip cage, non-infested sorghum had an increase change in reflectance while the infested plants displayed a comparatively decreased reflectance change. These reflection trends were seen across both the close and distal treatment groups.

Another interesting discovery was the difference between low and high aphid densities (T1 and T2). There is also a clear distinction as the lower aphid group (T1) had less reflection change than the higher aphid group (T2). There was a 0.2 predicted change in reflectance between low and high maps as seen in the darker blue colors in the high aphid versus low aphid. In addition, the largest difference between high and low aphid maps was between 500 and 650 nm range which is within the visible spectrum, specifically between green to red light wavelengths (Fig. 11). Specifically, the sorghum aphid spectral signature detected is between 550 and650 nm. Overall, the map in Fig. 11 shows a clear relationship between infested and non-infested plants and between low and high aphid densities.

## Discussion

This study provided additional insight about sorghum aphid and sorghum interactions and critical foundational information towards sensor-based UAS monitoring. Results in this study demonstrate a discernable spectral response in the visible range, 550–650 nm, between infested and non-infested sorghum with further distinction between low and high sorghum aphid densities. Additionally, these effects were statistically different for both proximity treatments, close and distal to the sorghum aphid active feeding site, showing a detectable local and systemic sorghum response. Our model system showed high predictability when identifying plants infested with aphids, with an average error of only 0.02 (Table 1); this level of confidence allows for highly accurate predictions of reflectance response to sorghum aphids is accurate. When more predictor variables are known, including period of sorghum aphid infestation (i.e., days post initial infestation), wavelength, aphid density, and proximity of sensor reading to active aphid feeding sites, then our accuracy in predicting spectral response for any plant in the system increases. Prediction of sorghum infestations over the 14-day experimental period allows for accurate predictions for every individual wavelength analyzed, which was 500–799 nm range, on any given sample date.

There was a clear distinction between sorghum infested or non-infested with sorghum aphid (Fig. 11). The most notable changes in reflectance values were observed in the visible green–red region of the electromagnetic spectrum or between 500 and 650 nm. This distinct wavelength range is where the most change in light reflectance occurred on sorghum leaves where sorghum aphids were present. Specific wavelength range changes in plants can provide insight about how stress, like aphid feeding, effects internal, physiological processes, which can lead to altered leaf structures and changes to how light is absorbed or reflected^12,16–18^. Spectral changes in the visible light range, 350–700 nm, is determined by changes in chlorophyll as blue and red light, about 400–500 nm and 600–700 nm respectively, is absorbed and green light, about 500–600 nm, is reflected^12,15,17,33–35^.

Therefore, most changes in reflectance in the visible range is likely due to changes in leaf chlorophyll content^15,17^. This correlation between changes in visible light and chlorophyll content corresponds with other studies, which show sorghum aphids causing decreased levels of chlorophyll, thus negatively impacting photosynthesis on susceptible sorghum varieties^36^. Continued chlorophyll degradation due to aphid feeding typically results in external leaf color changes such as chlorosis^15,37^. This means that sorghum aphid feeding causing changes in visible reflection will have a negative impact on chlorophyll and photosynthesis, thus causing leaves to yellow or reflect less green light.

Interestingly, sorghum aphid infestations can cause visible leaf discoloration but only at high population levels (GC personal observation^9^. This unique phenomenon allows sorghum aphid infested leaves at low aphid densities to remain greener longer^9^ than other sorghum feeding aphids, which cause relatively rapid leaf discoloration^38,39^. Paudyal et al. (2020)^36^ tested sorghum aphid density on susceptible sorghum and found that internal photosynthetic rates were impaired after only 72 h post infestation when 100 or more sorghum aphids were present but there were little to no external changes to the infested leaves. It is possible that extended feeding times and higher population levels are required to cause visible leaf damage. We were able to distinguish between high and low sorghum aphid densities (Fig. 11) but not the exact aphid number needed to elicit a plant response. If higher sorghum aphid populations are needed to cause external changes to sorghum leaves, this provides additional limitations to current sorghum aphid monitoring practices as it makes detection of low population densities more difficult to find. To reduce economic damage to sorghum, insecticide needs to be applied when sorghum aphid populations reach 50–125 aphids on 20–30% of plants^40^. This further justifies the need for sensors that can detect responses in sorghum to sorghum aphids before high population build up and cause visible leaf damage^19,41^.

Although changes in visible light correlate with a known decrease in chlorophyll content, and external leaf chlorosis at high population densities, our results show a decrease in reflectance in the green–red wavelength range due to sorghum aphid feeding. This was unexpected as a general plant response to stressors causes an increase in visible light and a decrease in NIR^12,42^. More specifically, plant stress causes an increase in red reflectance, due to decreased chlorophyll content and decreased absorption, and causes the reflectance peak of green light to widen^12,18,28,43^. Limited research has been conducted to assess spectral properties in sorghum to date^44^, but a few have shown similar trends in sorghum due to nitrogen (N) deficiency. Zhao et al. (2005) showed that nitrogen deficiency in sorghum caused an increase in green and red light, specifically around 555–715 nm, and a red-edge shift (Zhao et al. 2005). Singh et al. (2017) had similar findings in sweet sorghum as “nitrogen-sensitive wavebands” in the green and red region, specifically centered at 595–701 nm respectively, also increased due to changes in nitrogen^44^. However, exact plant spectral responses can vary between different aphid-crop systems^27,42,45,46^. Our findings showed a general decrease in reflectance between green and red wavelengths, 500–650 nm, but we could not discern specific wavelength changes within that range. Additional research is needed to understand why we saw decreased reflectance in the visible range, in both close and distal proximity treatments (Fig. 11), and how that could relate to internal sorghum aphid effects on sorghum.

Another component of our study looked at the sorghum spectral response at close and distal proximities to the aphid feeding site. Remarkably, similar sorghum responses were seen between close and distal locations, indicating that sorghum has a local and systemic response to sorghum aphid feeding. For our close proximity treatment, measurements were taken within a few centimeters of the site of infestation next to the clip cage, so we did not measure directly over the active feeding site. This indicates that sorghum aphids can elicit a discernable spectral response in nearby plant tissues. We hypothesize -aphids release saliva components that condition their host plant on a local level, such as impacting plant cell occlusion, and the impact of feeding can spread several centimeters from stylet penetration^47^. This provides a remarkable advantage to detecting leaf spectral response using hand-held spectrometers as aphid populations do not need to be removed from the leaf to take a spectral reading so they can continue to feed undisturbed.

Our predictive model also shows significant differences in spectral responses at sites distant from where aphids fed, which causes a systemic response by the plant that was observable using our light sensor. One possible explanation is that sorghum in this study responded through the induction of various defense pathways. For example, aphid feeding in general elicits the jasmonic and salicylic acid defense pathways that can be upregulated systemically throughout the plant^47,48^. However, an underlying question is whether upregulation of these defense responses cause enough internal changes to sorghum, due to sorghum aphid infestation, that causes the plant to absorb or reflect light differently than plants without aphids. Yang et al. (2009) observed that Russian wheat aphids and greenbugs feeding on wheat each elicited a distinct spectral response^28^ and that greenbugs on sorghum upregulated the jasmonic acid and salicylic acid defenses^29^. Based on these studies, it is plausible a similar mechanism explains the systemic response observed in this study; however, further investigation is needed to quantify such mechanisms using tissue extractions to quantity various plant constituents. The objective of the current study was to understand whether aphid feeding can elicit a response that is detectable using light reflectance and whether such a response could be observed on non-infested sorghum leaves.

### Broader research implications

The detection of both local and systemic sorghum response to sorghum aphid feeding is a critical find towards our main goal of using small UAS for more efficient field monitoring of this invasive species. For small UAS equipped with sophisticated sensors to accurately detect sorghum aphids, data from these devices need to be able to discern the presence of infestation regardless of feeding sites. Since a UAS captures images above the field canopy, measuring spectral changes is likely feasible since aphid feeding appears to be detectable in different parts of the plant. In other words, remotely sensed data from UAS do not capture reflectance of leaves deep in the canopy, which is where most sorghum aphids are found early in the colonization process^49^. Canopy distribution of sorghum populations are not uniform as sorghum aphids tend to feed on the bottom leaves resulting in a higher population density at the bottom of the canopy^50^. Uneven canopy distribution combined with a tendency to feed on the underside of leaves^1,49,51^, makes sorghum aphid outbreaks harder to spot. Further complications in monitoring for sorghum aphids are that high sorghum aphid populations are needed to cause visible changes to sorghum leaves, meaning that sorghum aphid populations have already exceeded economic threshold by the time visible signs of damage appear in sorghum^4,41^.

Our research tackles the practical application of using a UAS to detect sorghum aphids in an uneven canopy distribution. Sorghum was shown to respond locally and systemically to sorghum aphid feeding showing that sorghum aphid infestations on lower leaves can be detected from UAS readings on upper-canopy leaves. Traditionally, many remote sensing studies detecting sorghum aphid populations in sorghum using normalized differenced vegetation index (NDVI) but Lillesand and Kiefer (2000) found that use of NDVI is not always a reliable indicator as it cannot always differentiate between different plant stressors^52,53^. This further justifies our novel approach to analyzing sorghum’s spectral response as gradient boosted regression trees, as seen in the results (Table 1), is a very accurate method of predicting expected leaf reflectance in response to sorghum aphids. Regression trees also allow us to analyze sorghum’s response, change in reflectance, in relation to several variables including different starting densities of sorghum aphids, length of infestation, and proximity of spectral reading from sorghum aphid feeding site. Our analysis produced regression trees for every tested wavelength (i.e., 300 trees) within 500–799 nm range. To our knowledge, no current studies have used this machine learning approach in relation to an aphid-crop system or gained this level of detail between the multiple interactions associated with leaf spectral response and aphid infestation.

### Limitations of study

This study provides valuable information about sorghum aphid feeding on sorghum, which is a foundational study to future work involving remotely sensed data captured from autonomous vehicles. Future research should explore the mechanisms by which the plant is responding to infestation. In our study, we controlled water, nutrients, and other potential factors that could have affected plant growth and photosynthesis. Therefore, the models in the current study are only applicable to systems where such factors are controlled, and it is not appropriate to extrapolate these findings to field conditions. We also only tested one variety of sorghum, DKS 29–28, which is considered susceptible to sorghum aphids. However, there is a wide range of susceptible and resistant sorghum hybrids, with varied levels of antibiosis, antixenosis, and tolerance to sorghum aphids, that result in different responses to sorghum aphid feeding^54^. For instance, susceptible sorghum hybrids have been shown to have higher chlorophyll loss and faster rates of photosynthetic capacities decline compared to resistant hybrids under the same conditions^36^.

In addition to testing environment and plant variety, this study analyzed a limited wavelength range (500–799 nm), but the CI-710 Miniature Leaf Spectrometer has a range of 400–1000 nm. Since our data set was very large, we limited the wavelength range to regions of the spectrum where other studies have seen the highest sensitivity to stressors. Plant spectral responses to stress can vary but many plants show high sensitivity to stress between 535–640 nm and 685–700 nm^15^. For discernment of chlorophyll content, in relation to light reflection patterns, other studies found 530–630 nm (green–red light) and at the red-edge, around 700–750 nm^16,21,55–59^.

Lower wavelength ranges such as blue light, 400–500 nm, have overlapping absorption ranges between chlorophyll and carotenoid and are not recommended^60^. The blue range was also excluded due to technical issues with the CI-710 Miniature Leaf Spectrometer where we saw a lot of “noise” in the spectrometer output. Based on these studies, we limited our wavelength range to 500–799 nm to provide more confidence in the accuracy of the reflection readings and inclusion of the more spectrally sensitive wavelengths for detecting stress.

### Future directions

In this study we saw that sorghum aphids can be detected in sorghum by measuring leaf reflectance, both locally and systemically, and that changes in spectral responses are mostly observed between 500 and 650 nm. This allows us to focus our attention on the visible spectrum when using other remote sensing equipment, such as a UAS, to detect sorghum aphids in sorghum fields. We were able to distinguish spectral differences between non-infested, low-density, and high-density aphid groups but these values are based on aphid densities between all three experiments (R1, R2, and R3). The exact population number needed to elicit a detectable spectral response, and whether that number is below the 50-aphid threshold, remains unknown.

Although this is an important foundational study that adds additional insight into the sorghum aphid field monitoring, future research is needed to test the applicability of this study under different circumstances. This experiment was conducted under controlled conditions, with only sorghum aphid feeding, and testing only one susceptible variety of sorghum. In the field, other environmental stressors, such as drought, nutrient deficiency, or other insect infestations, would be present and detection of sorghum aphids when sorghum is undergoing multiple stressors is critical. For example, can sorghum aphid feeding be distinguished from other aphid pests such as greenbugs or yellow sorghum aphids? In addition, different sorghum hybrids, such as other susceptible and resistant varieties, are needed to see if the spectral response of infested sorghum in this study is a generalized sorghum response and not hybrid specific.

Looking into other factors, such as early or mature sorghum growth stages, are also needed to ensure that our results translate throughout sorghum development. On a physiological scale, the questions about what internal changes sorghum aphids cause sorghum, the unique decrease in visible reflectance under stress, would greatly increase our knowledge about how aphids impact plant responses to light. Overall, this is the first study to my knowledge that uses a “predictive approach” using boosted regression trees, to analyze a big data set measuring leaf spectral responses with high accuracy. In addition, this project showed additional information about sorghum aphid and sorghum relationships and provided new data concerning detecting these insect pests that brings us closer to developing a more efficient monitoring system.

## Data Availability

The dataset will be accessible upon request to the corresponding author following publication.
